# Therapeutic strategies for intracerebral hemorrhage

**DOI:** 10.3389/fneur.2022.1032343

**Published:** 2022-11-04

**Authors:** Zhe Li, Suliman Khan, Yang Liu, Ruixue Wei, V. Wee Yong, Mengzhou Xue

**Affiliations:** ^1^Department of Cerebrovascular Diseases, The Second Affiliated Hospital of Zhengzhou University, Zhengzhou, China; ^2^Academy of Medical Science, Zhengzhou University, Zhengzhou, China; ^3^Henan Medical Key Laboratory of Translational Cerebrovascular Diseases, Zhengzhou, China; ^4^Department of Clinical Neurosciences, Hotchkiss Brain Institute, University of Calgary, Calgary, AB, Canada

**Keywords:** intracerebral hemorrhage, secondary brain injury, therapeutic strategies, neuroinflammation, neuronal death

## Abstract

Stroke is the second highest cause of death globally, with an increasing incidence in developing countries. Intracerebral hemorrhage (ICH) accounts for 10–15% of all strokes. ICH is associated with poor neurological outcomes and high mortality due to the combination of primary and secondary injury. Fortunately, experimental therapies are available that may improve functional outcomes in patients with ICH. These therapies targeting secondary brain injury have attracted substantial attention in their translational potential. Here, we summarize recent advances in therapeutic strategies and directions for ICH and discuss the barriers and issues that need to be overcome to improve ICH prognosis.

## Introduction

Intracerebral hemorrhage (ICH) is a catastrophic stroke subtype with a high risk of disability and death. It represents 10–25% of all strokes and it afflicts an estimated 2 million people worldwide each year (1, 2). Effective treatment options for ICH are yet to be developed (3). Patients who survive ICH usually suffer from various neurological dysfunctions (4). Previous studies have shown that a variety of etiologies contribute to the development of ICH, among which hypertension is the most common cause (5). Other pathophysiologies such as amyloid angiopathy, brain tumors, aneurysms, arteriovenous malformations, cerebral cavernous malformations, and arteriovenous fistulas also give rise to ICH (6). A series of neuropathologic changes occur in the brain after ICH, including intracerebral hematoma, space-occupying effects due to secondary injury, changes in regional cerebral blood flow, brain edema and neurotoxic injury, and disruption of the blood-brain barrier (BBB) (7–10).

**Figure 1:**
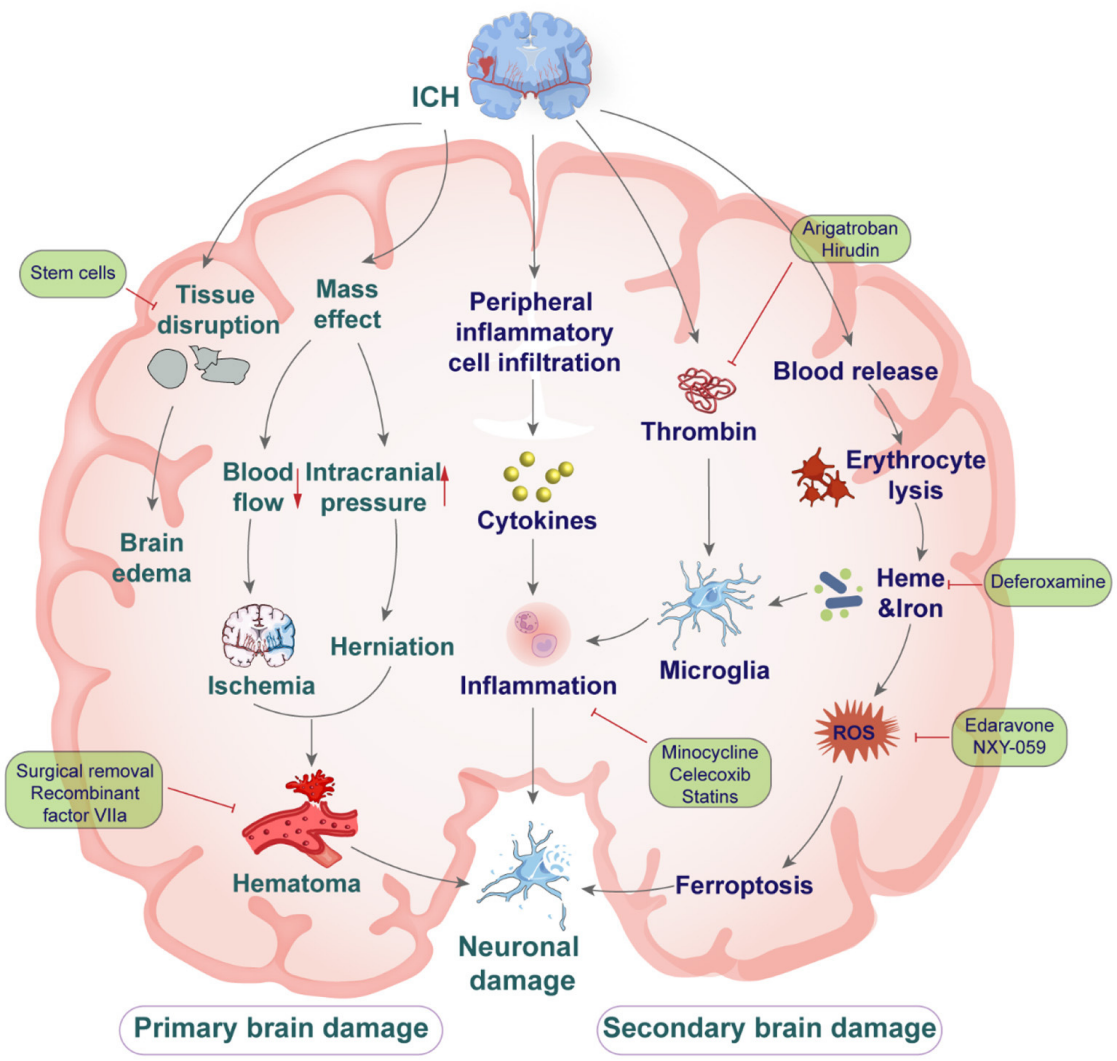
The mechanisms of primary and secondary brain injury after intracerebral hemorrhage, and drug intervention.

Complications of primary and secondary brain injury develop following ICH (11). Primary injury in the hyperacute phase of ICH is principally due to mechanical damage to the surrounding brain tissues as the hematoma develops (12, 13). The extent of primary injury is determined by the location and volume of the hematoma, and the initial hematoma size (14, 15). A consequence of hematoma formation and expansion is compression of the surrounding brain tissue causing neurological deterioration, intracranial hypertension, brain herniation and even death (16). Secondary brain injury after ICH involves a complex array of processes and consist of BBB damage, brain edema, iron deposition, cytotoxic cascade reaction, neuronal apoptosis, oxidative stress (17), and neuroinflammation (Figure 1) (18, 19).

It is important to explore potential therapeutic strategies that counter the mechanisms of primary and secondary brain injury after ICH (20, 21). In this review, the characteristics of pathophysiology following ICH are presented, as well as the current treatment options, and we emphasize the urgent need for investigating novel treatment strategies.

## Preclinical therapeutic strategies targeting ICH

In terms of etiology, intracerebral hemorrhage can be divided into three categories. Hypertension is the leading cause of ICH, a major focus of early treatment is to control blood pressure aggressively (15). A fast-acting agent such as nicardipine or clevidipine is the usual choice. Another common cause of ICH is cerebral amyloid angiopathy (CAA), patients with CAA are at high risk of recurrent hemorrhages and subsequent cognitive deterioration. Prevention of re-hemorrhage emphasizes avoidance of anticoagulation and antiplatelet, if possible, strict blood pressure control, and fall precautions (22). The remaining etiologies are due to secondary brain injury. The process of secondary brain injury is triggered by activation of resident cells and infiltration of peripheral immune cells, and inflammatory factors which contribute to the development of brain edema and cell death (23).

### Targeting anti-inflammatory responses

Brain injury after ICH occurs through multiple mechanisms including not only space-occupying effects of hematoma and mechanical damage, but also neuroinflammation (18). Neuroinflammation-related mediators include thrombin, matrix metalloproteinases, cytokines, free radicals, and complements. There is growing evidence that inflammation is one of the critical factors in secondary brain injury after ICH (20, 24, 25). The modulation of inflammatory responses in the early and delayed phases of ICH is particularly important, especially in the case of microglia/macrophages, astrocytes, and leukocytes (5, 18). Microglia are the initial and primary active immune defense fundamental to the CNS, which are thought to act as “the first responders” to brain injuries during the early stages of ICH (26). Microglia can undergo morphological and functional changes in response to disease signals. Microglia and monocyte-derived macrophages in the adjacent injured brain tissue of ICH are not readily distinguished from one another, thus we refer to them as microglia/macrophages (10, 27). In clinical studies, it has been shown that up-regulation of CD163 levels can facilitate microglia/macrophages phagocytosis (28); PPAR-γ agonist pioglitazone has been shown to be safe for hematoma regression after ICH (29); minocycline improves outcomes in patients with acute ischemic stroke, but efficacy and safety require further study (30). Preclinical research is oriented to regulate the function of microglia/macrophages. New therapeutic targets such as NLRP3 inflammasome (31), TSPO ectopic protein (32), and transforming growth factor TGF-β1 have been developed (33). Following ICH, astrocytes in the perihematomal area are activated and secrete various cytokines and chemokines, such as aquaporin AQP4 (34), IL-15 (35), IL-33 (36). Neutrophils can infiltrate around the hematoma within 4 h and release various inflammatory factors, which is an important predictor of neurological deterioration in primary ICH (37). In clinical trials, S1PR1 modulators, like fingolimod and RP101075, have shown to improve neurological deficits and promote recovery in patients with primary ICH (38). The cytokine IL-27, which accelerates neutrophil maturation in the bone marrow and reduces the secretion of proinflammatory cytokines, is also worthy of investigation as a potential therapeutic target for ICH (39).

Similarly, Toll-like receptor (TLR) activation and inflammatory signaling pathways regulated by risk-associated molecular patterns are engaged (40). Within 24 h after the onset of ICH, red blood cells are lysed and cytotoxic substances such as hemoglobin, heme and iron are released from the blood clot in the hematoma (24), which further exacerbate brain injury and ultimately lead to tissue damage, BBB dysfunction, and massive brain cell death (41, 42).

Numerous studies in animal models have shown that inhibition of neuroinflammation promotes recovery from ICH. A widely reported preclinical treatment strategy for ICH is the use of minocycline, which by inhibiting microglia and matrix metalloproteinase activation (25, 43–45), reduces brain edema, BBB damage, and brain cell death in collagenase induced ICH model in rat (46, 47). In addition, minocycline is a broad-spectrum tetracycline that may provide neuroprotection through its anti-inflammatory properties, including inhibition of microglia activity and alleviating demyelination in white matter after ICH (46, 48). We showed that minocycline exhibits protective roles in ICH by decreasing EMMPRIN and MMP-9 expression (49), alleviating BBB disruption, ameliorating neuroinflammation, and reducing neuronal degeneration and death (50, 51). Tuftsin fragment 1–3 has been found to attenuate ICH injury and improve neurological function by inhibiting microglia/macrophage activation in rats.

Clinical studies suggest that the anti-inflammatory effects of the cyclooxygenase inhibitors, celecoxib and pioglitazone, may be effective in lowering the neurological sequalae of ICH (52), but further studies are required to confirm this effectiveness. Similarly, statins that inhibit the synthesis of HMG-CoA reductase may not only have anti-inflammatory roles but also have a neuroprotective effect associated with increasing cerebral blood flow after ICH (53). Currently, two statins, rosuvastatin and simvastatin, have shown promising efficacy in clinical trials (54). While statins have encouraging short-term efficacy in the acute phase of ICH, there are no unequivocal clinical data to support the benefit of continued statin use after the acute phase. In addition, the risk of rebleeding that may result from long-term use of such drugs is unclear (55).

### Thrombin

Thrombin is a multifunctional serine protease that is rapidly produced after brain hemorrhage and catalyzes the conversion of fibrinogen to fibrin which affects the coagulation cascade. Thrombin may cause brain edema, neuroinflammation, and BBB damage in the early stages of ICH, but it is also essential to prevent initial hemorrhage and hematoma expansion (56). A dual effect of thrombin on blood vessels has been found. Low concentrations of thrombin cause vasodilation and exert neuroprotective effects by upregulating heat shock proteins and iron-related proteins. In contrast, at high concentrations, thrombin causes slow and persistent vasoconstriction, which leads to infiltration of inflammatory cells, disruption of the BBB, formation of brain edema, and neuronal death (24, 57). These findings suggest that inhibition of thrombin may reduce brain edema. Agatroban, a thrombin inhibitor, reduces brain edema around the hematoma after systemic treatment, while delaying secondary cerebral micro-thrombosis and improving local lateral cerebral blood flow. This observation suggests that thrombin inhibitors may provide a potentially useful neuroprotective strategy. In a similar manner, hirudin, another thrombin inhibitor, may reduce brain edema formation by downregulating aquaporin-4 and 9 in a collagenase-induced ICH model in rat (58–60).

### Antiplatelet therapy

More than 25% of patients with ICH have received antiplatelet therapy (APT), which may increase the risk of hematoma enlargement and lead to serious adverse events associated with ICH such as brain edema (42). It was investigated that long-term aspirin use increased in 12 cases of ICH per 10,000 people (61). Emergency treatment with low-dose aspirin (100 mg) within 48 h reduces the risk of recurrent stroke and improves prognosis (62). The risk of high-dose aspirin-induced ICH is increased in the elderly, especially in patients with untreated hypertension, and long-term combined use of aspirin and clopidogrel may elevate the risk of ICH (63).

A multicenter randomized controlled trial (PATCH) showed that patients receiving conventional platelet transfusions had a higher risk of post-ICH functional dependence or even death within 3 months, and therefore a higher chance of serious adverse events during hospitalization compared to the standard treatment group (64, 65). For the use of antiplatelet agents associated with ICH, it seems reasonable to avoid platelet transfusions. In prospective studies in patients with ICH, intravenous vasopressin improved platelet function without significant adverse effects, but to date, the effect of vasopressin on clinical outcomes has not been meaningfully evaluated (66).

### Complement inhibition

Current evidence suggests that the complement cascade is activated early after ICH and contributes to brain edema/injury in multiple ways (67). Complement cascade activation produces C3a and C5a, and microglia activation produces inflammatory mediators that subsequently assemble with complements into membrane attack complexes (MAC, C5b-9) on the surface of target cells (68). The formation of MAC may be associated with the release of hemoglobin and iron from erythrocyte lysis which exacerbate brain damage in the vicinity of the hematoma (14). Activation of the complement system is also a powerful driver of the activation of microglia and mast cells, thereby enhancing the inflammatory response of ICH (69).

Inhibition of complement cascade activation by means of antagonists or gene knockout can reduce the extent of brain injury. When ICH mice were treated with C5a-receptor antagonist, a reduction in neutrophil infiltration in the hemorrhagic hemisphere was shown at 24 and 72 h, and some neurological functions were improved. The combination of C5a-receptor antagonist and C3a-receptor antagonist led to synergistic improvements in the neurofunctional outcome while reducing inflammatory cell infiltration and brain edema (70).

However, experimental results on C5a remain controversial, with C5a knockout mice showing more severe brain damage after brain hemorrhage despite the protective effect provided by C5a-receptor antagonist, which may be related to compensatory changes in the organism's responsiveness after C5a loss (71). Therefore, given the role of the complement system in removing apoptotic cells and inducing neurogenesis, therapeutic complement inhibition needs to be delivered in a more targeted manner to maximize its efficacious effects while minimizing potential adverse effects (14).

### Improvement in iron chelator management

The process of hemolysis after ICH is featured by the release of erythrocyte contents, one of the main products of which is hemoglobin (Hb) which is then oxidized extracellularly to form oxygen radicals and free heme (72). Hb can also be degraded to ferrous iron, bilirubin and carbon monoxide by the action of heme oxygenase (73). Subsequently, due to the release of excess free iron ions from the hematoma, severe iron deposition in brain tissue may occur, triggering acute brain edema reactions and long-term neurocognitive impairment (74).

Basic studies and preclinical trials have shown that lowering brain iron deposits and reducing iron overload in brain tissue are reasonable strategies for the treatment of ICH. Deferoxamine based iron-lowering therapy significantly reduces brain edema induced by iron overload after ICH, attenuates neuronal death and improves neurological scores (75).

Deferoxamine through iron chelator markedly reduces free iron in brain tissue and is neuroprotective in a variety of animal models of ICH (76). In an autologous blood-induced ICH model in rats (77), deferoxamine attenuated brain edema and brain atrophy and improved neurological function, and these findings were corroborated in piglets (78). In addition, our recent study found that the combination of deferoxamine with minocycline provided better neuroprotection after ICH, with marked reduction in brain injury area, neuronal death and microglia/macrophage activation (79, 80). Preclinical data suggest that deferoxamine may prevent memory dysfunction by restoring iron homeostasis, attenuates neuroinflammation and oxidative stress (Table 1) (94). Meanwhile, clinical studies on deferoxamine are in phase II trials, but its safe dose and the emergence of late complications need to be further investigated (95, 96).

**Table 1:** Current and past therapeutics for intracerebral hemorrhage.

**Intervention**	**ICH type**	**Target**	**Phase**	**Outcome**	**Limitation**
Minocycline (81)	Primary ICH	Multiple	NCT01805895 Phases 1and 2	400 mg dose of minocycline achieves neuroprotective serum concentrations	Oral administration leads to delayed absorption, not suitable for severe patients
Celecoxib (82)	Primary ICH	COX-2	NCT00526214 Phase 2	Celecoxib limits the expansion of perihematomal edema in the acute phase of ICH	The 90 days functional outcome has not been significantly improved
Desmopressin (66)	Primary ICH	Antiplatelet	Phase II	Intravenous desmopressin was well-tolerated and improved platelet activity after acute intracerebral hemorrhage	Larger studies are needed to determine its potential effects on reducing hematoma growth vs. platelet transfusion or placebo
Fingolimod (38)	Primary supratentorial ICH	Neuroinflammation	Phase II	Administration of oral fingolimod reduced PHE, attenuated neurologic deficits, and promoted recovery	The efficacy of fingolimod in preventing secondary brain injury in patients with ICH warrants further investigation in late-phase trials
Deferoxamine Mesylate (83)	Spontaneous supratentorial ICH	Iron	NCT02175225 Phase 2	DFO can effectively improve nerve function, and the dose of 32 mg/kg/day is safe	Screening of patients with ICH has slight limitations, and the generalizability of the results needs to be considered
Factor VIIa (84)	Acute ICH	Hematoma expansion	NCT00127283 phase 3	Significantly reduced growth of the hematoma	Did not improve survival or functional outcome at 90 days
STICH II (85)	Spontaneous lobar intracerebral hematoma	Surgical evacuation	ISRCTN22153967	Early surgery has clinically relevant survival advantages and does not increase mortality or disability at 6 months	Prospective randomized controlled trials have not been conducted to compare the effect with conservative treatment
PATCH (64)	Supratentorial ICH	Surgical evacuation	NTR1303	Compared to the standard treatment group, patients who routinely receive platelet transfusions have a higher probability of functional dependence at 3 months	1. The sample size was smaller 2. The level of bias through selective inclusion is unknown 3. Adherence to antiplatelet therapy for participants was not measured
MISTIE III (86)	Supratentorial ICH	Surgical evacuation	NCT01827046	Minimally invasive treatment is safe and helps reduce the fatality rate at 365 days	Does not improve nerve function, need to be confirmed in the future
RP101075 (87)	Autologous blood/mouse	S1PR1 agonist	Pre-clinical	RP101075 significantly attenuated neurological deficits and reduced brain edema in ICH mice	Provides insufficient information on the optimal S1PR1 modulation time window
VK-28 (88)	Autologous blood or collagenase/mouse	Iron	Pre-clinical	VK-28 decreased iron-deposition and microglial activation around hematoma, and improved neurologic function	The dose–response and the therapeutic window need to be determined in young and aged animals
Argatroban (89)	Autologous blood or collagenase/rat	Thrombin	Pre-clinical	Systemic administration of argatroban after 6 h can also reduce the formation of ICH-induced edema	Argatroban used in clinical trials may have a higher potential risk of rebleeding
Stem-cell transplantation (90)	Collagenase/rat	Multiple	Pre-clinical	BM-MSCs transplantation reduced hematoma volume and alleviated neurological deficits after ICH	Further research is needed to explore the effect of BM-MSCs in later stages of this condition
Therapeutic hypothermia (91)	Collagenase/rat	Multiple	Pre-clinical	Prolonged mild hypothermia provides persistent histologic and functional protection	Early hypothermia brings complications, such as elevated blood pressure and coagulopathy
TAK-242 (92)	Autologous blood/mouse	TLR4	Pre-clinical	TLR4 antagonist reduced inflammatory injury and neurological deficits, decreased DNA damage and neuronal degeneration	Need to be further verified in clinical trials
PD-1 (93)	Collagenase/mouse	Multiple	Pre-clinical	Significantly attenuated neurological deficits, reduced brain edema, and decreased hemorrhage volume	No direct evidence for the influence of PD-L1 on the apoptosis or proliferation of neurons and astrocytes

### Neuroprotective agents

Neuroprotective agent therapy has been investigated in the acute and chronic stages of ICH. Valproic acid (VPA), an anticonvulsant, may have neuroprotective roles after ICH. VPA was found to enhance the expression of the anti-apoptotic gene Bcl-2, which limits the development of hematoma by inducing the extracellular signal-regulated kinase cAMP response element-binding protein after ICH (97). In addition, VPA not only down-regulates the expression of pro-inflammatory factors such as MMP-9 and FasL, but also acts as an anti-apoptotic neuroprotective agent by inhibiting the activation of Caspase-3. Recent studies have shown that VPA can protect rat cortical neurons from glutamate-induced excitotoxicity and increase the lifespan of cultured cortical neurons (98).

Various endogenous proteins are upregulated to protect the brain from various mechanisms of injury. Nuclear factor erythroid 2-related factor 2 (Nrf2) is a transcription factor that regulates antioxidant defense mechanisms by responding to oxidative stress (99). Nrf2 knockout mice were more susceptible to oxidative stress than wild-type mice. The apoptosis and neurological dysfunction in collagenase-induced ICH model were more severe in Nrf2 knockout mice compare to wildtype mice (100). Erythropoietin (EPO) is a glycoprotein hormone that is an important regulator of cell function, survival, and various molecular cascade responses (101). It is involved in STAT-3 and eNOS pathways and can down-regulate glutamate excitotoxicity, apoptosis and inflammation, thus exerting neuroprotective and anti-apoptotic effects (102). Similarly, programmed cell death 1 (PD-1) represents an important immune receptor that is expressed on a variety of activated immune cells. Related studies have demonstrated that PD-1 inhibits inflammatory cell activation, reduces neurological deficits, hematoma volume and neuronal death in mice, and enhances BBB integrity to a certain degree (93).

## Clinical therapeutic strategies targeting ICH

### Blood pressure management

Patients with ICH often have elevated blood pressure, possibly due to stress, pain, or high cranial pressure. Hypertension after ICH is often associated with hematoma enlargement and poor prognosis, but the relationship is unclear (12). The treatment of hypertension is currently in a dilemma. On the one hand, the presence of hypertension increases the risk of edema, which may lead to persistent bleeding and rebleeding causing exacerbation. On the other hand, the occurrence of hypertension may be a protective mechanism to ensure cerebral blood flow supply and prevent ischemic stroke (103). Aggressive antihypertensive therapy after ICH can easily lead to a sudden drop in cerebral perfusion pressure and cerebral ischemia, followed by increased intracranial pressure and further neurological impairment.

Guidelines from the American Heart Association and the Stroke Association state that in patients with systolic blood pressure between 150 and 220 mmHg who do not have a contraindication to acute blood pressure lowering, continuous use of antihypertensive agents such as labetalol, esmolol, or nicardipine should be used. It is safe to reduce blood pressure to 130–140 mmHg within a couple of hours, but its effectiveness in improving neurological function remains to be further examined (104). In patients with ICH with systolic blood pressure >220 mmHg, blood pressure can be controlled by continuous intravenous drug infusion with a target systolic blood pressure of 160 mmHg with close monitoring of blood pressure. In the presence of impaired blood flow autoregulation, mean arterial pressure decreases by more than 15–30%, which may exacerbate ischemia in the area surrounding the hematoma and worsen brain injury. Notably, intensive hypotension may lead to adverse renal events (105).

### Hematoma expansion

Hematoma expansion has a significant relationship with clinical deterioration and poor prognosis. To date, prevention of hematoma expansion is an effective strategy to reduce the mass occupying effect. Traditionally, hypertension is considered to be one of the main causes of ICH; indeed, persistent elevation of systolic blood pressure is one of the main risk factors for hematoma expansion. Clinical trials have indicated that there is no significant difference in the elevation of systolic blood pressure between the hematoma-dilated and non-hematoma-dilated groups of patients with ICH (73, 106), and that elevation of systolic blood pressure is not an independent factor for hematoma expansion in the acute phase of a ICH when studied with multivariate analysis, but that rapid reduction of blood pressure may contribute to improved functional outcomes (107).

A further approach to prevent hematoma expansion is the use of drugs that alter the coagulation cascade or fibrinolytic process. The results of phase II clinical trial of recombinant factor VIIa show that treatment within 4 h after the onset of ICH limited hematoma expansion and improved clinical regression, but the incidence of thromboembolic events was mildly increased (84). E-aminocaproic acid (EACA) is an antifibrinolytic agent that helps to limit hematoma volume expansion and reduce early morbidity and mortality, but the long-term benefit is uncertain (108). Therefore, the focus of future studies should be to determine which subgroup of patients would benefit from activated recombinant factor VIIa therapy (63, 65). Clinical trials have demonstrated the benefit of early platelet transfusion in patients with the ICH who have received antiplatelet therapy, while in thrombolytic drug-related ICH, transfusion of coagulation factors and platelets may be an option (109).

## Surgical therapeutic strategies targeting ICH

After vascular rupture, a hematoma begins to form and develops within 60 min, causing mechanical damage to the brain parenchyma. The hematoma first causes deformation and displacement of brain tissue, which in turn increases intracranial pressure and elevates the risk of brain herniation and cerebral ischemia (55). Many clinical trials have explored and tested the effectiveness of clot removal and focused on reducing surgical complications (110).

### Craniectomy for evacuation of hematoma

Craniotomy is the initial and main method of neurosurgical treatment of ICH. Early removal of hematoma can reduce hematological toxicity, alleviate edema and ischemia around the hematoma, prevent hematoma expansion, and produce a good clinical treatment effect (111). The surgery allows for direct vision, adequate decompression, and precise hemostasis, which not only reduces intracranial pressure but also improves hemodynamics and brain tissue metabolism. However, at the same time, craniotomy causes greater surgical trauma and a higher incidence of postoperative complications, which may have serious prognostic implications (112, 113).

In a multicenter randomized controlled trial, early surgical manipulation was not significantly beneficial to patients compared to conservative treatment (114). In the subsequent STICH II study, in patients with spontaneous superficial ICH without ventricular hemorrhage, early surgical resection of lobar hemorrhage did not result in a better clinical outcome and produced only a slight survival advantage compared with pharmacological treatment (85). Regarding cerebellar hemorrhage, surgical treatment has a better prognosis for patients whose condition deteriorates rapidly due to brainstem compression or hydrocephalus. In patients with spontaneous ICH without ventricular hemorrhage, early surgery may have a small but clinically meaningful survival advantage (115).

### Minimally invasive surgery

The application of minimally invasive surgery (MIS) to obtain maximum results at the cost of minimal trauma has helped to improve the surgical outcome of ICH. The application of various minimally invasive techniques has become a mainstream concept worldwide. The most commonly used techniques include hematoma directed drainage, keyhole surgery, and neuro-endoscopic hematoma evacuation (116). The advantages of minimally invasive surgery for ICH are reduced surgical exposure, shorter operative time, less surgical trauma, and the procedure can be performed under local anesthesia.

Meta-analysis of a clinical trial found better treatment outcomes, improved neurological recovery, and reduced long-term mortality with minimally invasive surgical treatment compared to other treatments (conservative and craniotomy) (114).

Further studies have found that minimally invasive surgery combined with thrombolytic therapy within 72 h after ICH is safe, reduces perifocal edema, promotes hematoma liquefaction and drainage *via* catheter, and has a tendency to improve neurological function (117, 118). It is worth noting that minimally invasive surgery is less effective in decompression and is not superior to conservative medical therapy in terms of CNS infection rate and symptomatic rebleeding. Therefore, patients with ICH of undetermined etiology should undergo vascular-related investigations (CT angiography, MR angiography and digital subtraction angiography) prior to minimally invasive surgery to exclude vascular lesions and avoid and reduce the risk of rebleeding (119).

### Extraventricular drainage

Intraventricular hemorrhage (IVH) varies in severity and is a common complication of ICH and an independent assessment factor of ICH prognosis. IVH often leads to acute hydrocephalus, which can be treated by lumbar puncture or external ventricular drainage (EVD) (113). In recent years, several studies have shown that fibrinolytic drugs can be used as an adjunct to extraventricular drainage and that their combination can prevent drainage obstruction. CLEAR III, a large multicenter randomized controlled study that included 500 patients, found that EVD combined with rt-PA cleared ventricular hemorrhage with good safety and helped reduce mortality in patients with severe ventricular hemorrhage, but neurological function was not improved. Improvement in neurological function did not occur, and further studies are needed (111, 120). According to another study, EVD + rt-PA combined with lumbar puncture and drainage helped to clear ventricular hemorrhage more rapidly, and the risk of subsequent ventriculoperitoneal shunts and rebleeding was significantly reduced; a follow-up randomized clinical trial (RCT) is still needed to verify this (116).

## Emerging therapeutic strategies targeting ICH

### Stem cell therapy

Stem cell therapy has emerged as a potential approach for the treatment of ICH, mainly to modulate the immune response after ICH. The replacement of damaged cells and restoration of function is accomplished by transplanting cells such as mesenchymal stem cells, neural stem cells, embryonic stem cells, stem cells from bone marrow and umbilical cord blood (12). Mesenchymal stem cells (MSC) are umbilical cord-derived cells with powerful immunomodulatory capabilities, and intracerebroventricular transplantation of MSC can prevent further development of brain injury and hydrocephalus after ICH (90). Bone marrow mesenchymal stem cells (BMSC) secrete neurotrophic factors that promote astrocyte proliferation and myelin formation by intravenous injection and improve neurological and behavioral performance in rats with collagenase-induced ICH (121, 122). Recently, human umbilical cord blood (HUCB) has been considered as an ideal cell source for the treatment of ICH. In a collagenase-induced rat ICH model, human umbilical cord blood and its derived single nucleated cell transplantation reduced hematoma volume and improved functional recovery (90, 123).

#### Potential MicroRNA therapy

MicroRNAs (miRNA, miR) are small conserved non-coding single-stranded RNA. They can regulate inflammatory response after ICH and are viable molecular targets to alter brain function (124, 125). Over the past few years, there has been a surge of research addressing the role of various miRs in the pathophysiology of ICH (126). For example, several studies have demonstrated that miR-223 (127), miR-7 (128), miR-let-7a (129), miR-23b (130) and miR-194-5p (131), among others, have roles in promoting neuroprotection. Given the potential of miR as a viable therapeutic target, several clinical trials are already underway. One clinical trial assesses the efficacy of exosomes overexpressing miR-124 in improving the outcome from acute ischemic stroke (NCT03384433; clinicaltrials.gov) (132). Further studies are required to elucidate the molecular mechanisms of miR dysregulation after ICH and to test the safety and tolerability of miR-based therapeutic strategies.

#### Nanotechnology

Nanotechnology has been widely used for disease monitoring, diagnosis, and for the treatment of ICH. Moreover, drug delivery strategies that can bypass the BBB physical barrier have benefited from polymer-based nanoparticles (NPs) due to their desirable biocompatibility and ability to improve the bioavailability and pharmacokinetics of specific drugs.

In one study, curcumin Cur was encapsulated in NPs (Cur-NPs), and these Cur-NPs effectively crossed the BBB to enhance the accumulation of curcumin in the brain (133). More importantly, Cur-NPs have the ability to inhibit ferritin formation and thus can be used as an effective treatment for ICH. A similar study combined the covalently bonded iron chelator deferoxamine (DEF) with a carbon nanomaterial, polyethylene glycol-conjugated hydrophilic carbon cluster (PEG-HCC), which was synthesized as a multifunctional nanoparticle of antioxidant with iron chelator property; in addition, DEF-HCC-PEG protected cells from senescence and iron atrophy, and protected neurons from the toxic effects of blood breakdown products in ICH (134).

### Therapeutic hypothermia

Hypothermia is an effective neuroprotective agent in rodent models of ischemic stroke (135). It prevents neuronal loss and improves functional outcomes (136). Studies have shown that hypothermia can also reduce mortality and promote neurological recovery in patients with cardiac arrest (137, 138). In a rat whole-blood ICH model, early hypothermia (1 h after injection) resulted in reduced edema but no significant improvement in neurological outcomes, whereas delayed hypothermia (12 h after injection) showed improvement in neurological outcomes. Experts speculate that this is due to early hypothermia leading to impaired coagulation and hypertension, which exacerbates hematoma expansion (139).

Data from clinical studies have shown that patients with ICH treated with sub-cold temperatures (brain temperature of 30–35°C) for 8–10 days have reduced peri-hematoma edema and neutrophil infiltration, improved DNA damage and neurological deficits, and reduced mortality. More than 90% of mild hypothermia treated patients exhibited pneumonia as a side effect but this responds to intravenous antibiotics (140). The Safety and Feasibility Study of Targeted Temperature Management after ICH (TTM-ICH) is currently underway and is expected to provide additional guidance (91, 137).

### Neuro-critical care management

Patients with ICH are typically treated in general intensive care units, while studies have shown that managing ICH patients within a neurocritical care system has a more positive impact on their prognosis and may influence clinical endpoints (141). Patients with ICH can benefit from an early and aggressive treatment plan, with care focused on neurological monitoring, treatment of elevated intracranial pressure or cerebral ischemia, and prevention and treatment of secondary medical complications, which should be performed by experienced nurses, physicians, and other medical staff. Kurtz et al. worked on the management of patients with brain injury in specialized and general ICUs; they highlight differences in indicators such as hemodynamics, intracranial pressure monitoring, tracheotomy, nutritional support and intravenous sedation doses in the NICU (142). Another study found that patients in general ICUs had a 20–40% higher in-hospital mortality rate compared to neurological ICUs (143). The highest priority in the treatment of patients with brain injury is the avoidance of secondary brain injury, so effective management of disease problems and risks, better adherence to established clinical guidelines, more careful monitoring, and more aggressive interventions can lead to a better prognosis for patients with ICH.

## Review and outlook

ICH has attracted worldwide attention as a disease that decreases the quality of life of survivors. It is often life-threatening. In the last 20 years, the number of preclinical and clinical studies on ICH has increased considerably and this has led to a better understanding of the mechanisms of injury and potential therapeutic targets for ICH. However, exploring the appropriate and effective treatment method remains a challenge (20).

Patients with ICH require the following measures to be treated effectively: identification of the underlying cause of the hemorrhage, correcting elevated blood pressure, reversing coagulopathy, and providing supportive care in a neurological ICU. Clinical experience shows that the major prognostic interventions after ICH are ultra-early hemostasis, clot evacuation, and intracranial pressure control. Therefore, we need a better understanding of the dynamic process of hematoma growth and to minimize the inflammation caused by hemorrhage and the neurotoxicity produced by blood degradation products (144). Related treatments include ultra-early hemostatic therapy with activated recombinant factor VIIa, acute treatment of hypertension, tight glucose control, and the management of coagulation disorders. Other innovative techniques, such as thrombolytic treatment for ICH, minimally invasive surgery, the development of new anti-inflammatory medications, and the introduction of neuroprotective agents, need further study (e.g., deferoxamine, statins, celecoxib, pioglitazone, etc.). Clinical trials are also actively advancing, and new therapies may be developed in the near future. Other strategies such as hypothermia, neural stem cell transplantation, and professional surgical care, are also expected to contribute additional advances in the coming years (12, 145).

However, the treatment of ICH still faces major problems and obstacles. First, we do not fully understand the pathological mechanism of secondary injury after ICH and the complex molecular pathways leading to brain injury, which limits the development of therapeutic strategies. Although various studies have addressed gene expression and various molecular pathways, the following questions are not entirely clear, for example, whether there is an association between reduced brain edema volume and improved neurological function, and whether the hypometabolism and hypoperfusion surrounding the hematoma is associated with neuronal death (146, 147).

Second, animal models of ICH used in basic research have their own advantages and disadvantages, but none of them fully replicates human ICH; therefore, it is necessary to select an appropriate animal model according to the purpose and conditions of preclinical studies and to critically evaluate its results. Finally, the vast majority of ongoing investigations are still in the preliminary experimental phase and have not advanced to clinical trials, thus the distinctions between the two are not yet well-defined. Therefore, careful consideration should be given to study design, measurement of outcomes, optimal timing of intervention, and heterogeneity among subjects (6, 148).

## Conclusions

In summary, the optimal treatment strategy for ICH is effective prevention and long-term supervision for high-risk groups, internal medicine support and systemic treatment, surgical removal of hematoma with minimal trauma, and early rehabilitation by a variety of rehabilitation therapies (12). Although ICH poses a tremendous burden on the world's public health, much relevant research has been done to address the problem and we remain cautiously optimistic on the prospects of effective ICH treatment (147).

## Author contributions

Conceptualization, formal analysis, and writing—original draft: ZL. Writing—review and editing: ZL, MX, VY, SK, YL, and RW. Supervision: MX and VY. All authors have read and agreed to the published version of the manuscript.

## Funding

The authors acknowledge operating grant support from National Key Research and Development Program of China (grant no. 2018YFC1312200), National Natural Science Foundation of China (grant nos. 82071331, 81870942, and 81520108011), China Postdoctoral Science Foundation (grant nos. 2020TQ0289 and 2020M672291), and from the Canadian Institutes of Health Sciences (VY).

## Conflict of interest

The authors declare that the research was conducted in the absence of any commercial or financial relationships that could be construed as a potential conflict of interest.

## Publisher's note

All claims expressed in this article are solely those of the authors and do not necessarily represent those of their affiliated organizations, or those of the publisher, the editors and the reviewers. Any product that may be evaluated in this article, or claim that may be made by its manufacturer, is not guaranteed or endorsed by the publisher.
